# Male Seminal Relaxin Contributes to Induction of the Post-mating Cytokine Response in the Female Mouse Uterus

**DOI:** 10.3389/fphys.2017.00422

**Published:** 2017-06-19

**Authors:** Danielle J. Glynn, Kee Heng, Darryl L. Russell, David J. Sharkey, Sarah A. Robertson, Ravinder Anand-Ivell, Richard Ivell

**Affiliations:** ^1^Robinson Research Institute and School of Biological Sciences, University of AdelaideAdelaide, SA, Australia; ^2^Robinson Research Institute and Adelaide Medical School, University of AdelaideAdelaide, SA, Australia; ^3^School of Pharmacy and Medical Sciences, University of South AustraliaAdelaide, SA, Australia; ^4^School of Biosciences, University of NottinghamUnited Kingdom

**Keywords:** seminal relaxin, cytokines, endometrium

## Abstract

The hormone relaxin is important in female reproduction for embryo implantation, cardiovascular function, and during labor and lactation. Relaxin is also synthesized in males by organs of the male tract. We hypothesized that relaxin might be one component of seminal plasma responsible for eliciting the female cytokine response induced in the uterus at mating. When recombinant relaxin was injected into the uterus of wild-type (*Rln*^+/+^) mice at estrus, it evoked the production of *Cxcl1* mRNA and its secreted protein product CXCL1 in four of eight animals. Mating experiments were then conducted using mice with a null mutation in the relaxin gene (*Rln*^−/−^ mice). qRT-PCR analysis of mRNA expression in wild-type females showed diminished uterine expression of several cytokine and chemokine genes in the absence of male relaxin. Similar differences were also noted comparing *Rln*^−/−^ and *Rln*^+/+^ females mated to wild-type males. Quantification of uterine luminal fluid cytokine content confirmed that male relaxin provokes the production of CXCL10 and CSF3 in *Rln*^+/+^ females. Differences were also seen comparing *Rln*^−/−^ and *Rln*^+/+^ females mated with *Rln*^−/−^ males for CXCL1, CSF3, and CCL5, implying that endogenous relaxin in females might prime the uterus to respond appropriately to seminal fluid at coitus. Finally, pan-leukocyte CD45 mRNA was increased in wild-type matings compared to other combinations, implying that male and female relaxin may trigger leukocyte expansion in the uterus. We conclude that male and/or female relaxin may be important in activating the uterine cytokine/chemokine network required to initiate maternal immune adaptation to pregnancy.

## Introduction

In the female mammal the peptide hormone relaxin is secreted into the circulation principally from the ovary and, during pregnancy, probably also from the endometrium and placenta (Sherwood, 1994; Ivell and Einspanier, 2002). It may also be expressed as a paracrine hormone in some organs, including the heart (Taylor and Clark, 1994). Its principal functions in the context of female reproductive physiology are associated with ovulation (Brannström and MacLennan, 1993), implantation and endometrial decidualization (Ivell and Einspanier, 2002; Bartsch et al., 2004), placental growth and angiogenesis (Parry and Vodstrcil, 2007; Einspanier et al., 2009), myometrial quiescence in some species associated particularly with term of pregnancy (Downing and Sherwood, 1985; MacLennan et al., 1986), but also with spacing of embryos in multiparous rodents (Rogers et al., 1983). More recently, attention has focused on the role of relaxin in cardiovascular physiology both during pregnancy and in the non-pregnant state, where it appears to act in a vasodilatory role and to reduce arterial resistance (Conrad, 2011). Exogenously applied relaxin appears to be beneficial for patients suffering acute heart failure (Ponikowski et al., 2012).

Far less is known about relaxin in the male (summarized in Ivell et al., 2011), except that it appears to be produced in moderate amounts by the prostate gland in several species (Gunnersen et al., 1995; Samuel et al., 2003), and in some also within the seminal vesicles (Kohsaka et al., 1992). In terms of functionality in the male, paracrine roles have been suggested within the prostate gland and in the context of prostate carcinoma (Ivell et al., 2011). There is also now some evidence to suggest that relaxin may act directly on spermatozoa, thereby improving fertilization rates, at least *in vitro* (Miah et al., 2006; Ferlin et al., 2012).

For a number of years it has been known that relaxin can act on the human monocytic cell-line, THP1 (Parsell et al., 1996; Anand-Ivell et al., 2007), and also on primary decidual macrophages (Horton et al., 2011), as well as macrophages produced by differentiating THP1 cells *in vitro* using phorbol-myristate-acetate (Anand-Ivell and Ivell, 2014). Additionally, there are studies suggesting a specific role of relaxin on uterine lymphocytes (Piccinni et al., 1999, 2000). Relaxin may also act on cervical cells as well as on the term amniotic membrane, partly to cause the stimulation and secretion of some cytokines (Millar et al., 2003; Horton et al., 2011), which may assist in the birth process and peripartal recovery. Thus, relaxin may have functional properties which influence the immune response, though a systematic study of an immunological role for relaxin is lacking.

There is growing evidence to suggest that during copulation, besides spermatozoa, seminal plasma also delivers a range of signaling molecules into the female tract (Robertson et al., 2006; Robertson, 2007; Bromfield, 2014). One of the most important of these is TGFβ (Sharkey et al., 2012a), though other molecules, including prostaglandins are likely to be involved. The main role for such seminal plasma components appears to be to elicit an appropriate immunological response in the uterus so that on the one hand unsuccessful sperm can be quickly removed by phagocytosis, but also that newly formed semi-allogenic embryos are not rejected by the maternal immune system (Robertson, 2007, 2010). This immune response is initiated when uterine epithelial cells respond to TGFβ and other active signaling factors in semen to upregulate expression of several cytokines and chemokines (Robertson et al., 1992, 1998). In turn, these recruit macrophages, granulocytes, and dendritic cells (Kachkache et al., 1991; McMaster et al., 1992) which then activate regulatory T cells to mediate maternal immune tolerance at the time of embryo implantation (Johansson et al., 2004; Robertson et al., 2009).

The components of seminal plasma are mostly generated by the epithelia of the prostate gland and/or the seminal vesicles, though some molecules will also be transported from the epididymis and testis. Relaxin has been shown to be present in moderate concentration (0.2–2.0 nM) in the seminal plasma of most mammals (Ivell et al., 2011). Relaxin immunoreactivity as well as evidence for the presence of the specific relaxin receptor, RXFP1, also suggest the possibility that relaxin may be additionally present on spermatozoa (Carrell et al., 1995; Feugang et al., 2015; Almeida et al., 2016). However, it remains to be determined whether relaxin of male origin has the capacity to influence uterine function at the time of insemination. Here we have employed two mouse models to evaluate this. Firstly, estrous virgin wild-type female mice received human recombinant relaxin or vehicle by injection into the exteriorized dorsal horns of the non-pregnant uterus, with uterine tissue as well as luminal lavage fluid being collected shortly thereafter and analyzed. Secondly, we used mice with a null mutation in the gene encoding relaxin (*Rln*^−/−^ mice) to devise controlled matings between wild-type and homozygous null mutant males and females in all combinations, followed again by analysis of uterine tissue and uterine fluid lavage. There is no a priori evidence to suggest any implantation defect in these mice and litter sizes appear normal (Zhao et al., 1999). The results of these experimental approaches strongly suggest that relaxin of male (and also female) origin has a significant effect in the female reproductive tract, eliciting uterine production of pro-inflammatory cytokines at the time of insemination and fertilization.

## Materials and methods

### Animals and treatments

Experimental mice were generated from breeding pairs heterozygous for the *Rln* null mutation (*Rln*^+/−^ mice) at the University of Adelaide from founders generously provided by Dr. Chrishan Samuel (Melbourne, Australia), as previously described in Zhao et al. (1999). All mice were genotyped as *Rln*^−/−^, *Rln*^+/−^, or *Rln*^+/+^ from tail clip DNA extracted by the method of Laird et al. (1991), using the PCR primers and conditions as described (Zhao et al., 1999).

In the first experiment, wild type (*Rln*^+/+^) adult (8–12 weeks) virgin female mice in estrus were identified by daily vaginal smearing (Snell, 1941). Mice were anesthetized with isoflurane, and each uterine horn exteriorized dorsally through an incision in the body wall. Using an insulin syringe, 15 μl of either vehicle alone (RPMI with 0.1% BSA) or vehicle plus 25 ng human recombinant relaxin (rhRLN; a generous gift from Professor John Wade, Melbourne; this peptide was prepared and validated as in Stults et al. (1990) and shown by mass spectrometry to be >98% pure) were injected successively into each uterine horn, before replacing the tract, clipping the wound, and allowing recovery on a heated pad. rhRLN has been shown to have full bioactivity with even slightly lower EC50 compared to rat relaxin in a mouse RXFP1 receptor bioassay (Scott et al., 2004), having high homology in those domains responsible for receptor binding (Bathgate et al., 2006). Thus, altogether relaxin-treated mice received a total of 50 ng rhRLN in 30 μl vehicle per uterus, equivalent to ~270 nM which, accounting for degradation and dilution, should still be more than sufficient to activate murine RXFP1 receptors (EC50 ca 1 nM; Samuel et al., 2007). Although mouse seminal relaxin concentration is not known, data from other species suggest a seminal concentration of ~0.2–2 nM (Ivell et al., 2011). After 10 h, a time similar to previous studies (Schjenken et al., 2015), mice were killed by cervical dislocation and uteri dissected. Each horn was flushed with 250 μl RPMI with 0.1%BSA, the top quarter of one horn was immediately frozen in liquid nitrogen for later RNA extraction, and the remaining uterine tissue was frozen in OCT medium for immunohistochemistry.

In the second experiment, adult virgin females (*Rln*^−/−^ and *Rln*^+/+^) were mated overnight with either *Rln*^−/−^ or *Rln*^+/+^ males of proven fertility. After checking for copulatory plugs the following morning, the females were killed between 10:00 and 12:00 h by cervical dislocation. Uterine lavage and uterine tissue were collected as above.

Additionally, adult male wild type mice (Dummerstorf DUK strain) were euthanized as above and testes, epididymides, seminal vesicles, vas deferens, prostate, preputial, and coagulating glands were dissected for extraction of RNA (see below). Also testes and epididymides from adult sibling wild type (+/+) and relaxin deleted (−/−) male mice were similarly processed. Animal experimentation was approved by the Animal Ethics Committee of the University of Adelaide (approval number S-042-2007) or by the Animal Ethics Committee of Mecklenburg-Vorpommern, Germany (no. LALLF M-V/TSD/7221.3-1.1-033/06).

### Analysis of uterine tissue

Rat anti-mouse mAb hybridoma supernatants recognizing CD45 (TIB122; reactive with all leukocytes) and Ly-6G (RB6-8C5; reactive with neutrophils) were used to determine the density and distribution of leukocytes within the mouse endometrium (Supplementary Table 1). Cross-sections of uterine tissue (7 μm) were cut from OCT-embedded frozen uterus, air-dried, and stored at −80°C with silica gel (Ajax Finechem, Auburn, NSW, Australia) until staining was performed. Sections of uterine tissue were fixed in 96% ethanol for 10 min at 4°C, then rinsed three times in PBS. Non-specific binding was blocked by incubation with 1% (w/v) BSA in PBS for 2 min. Duplicate non-serial sections were incubated with neat hybridoma supernatant (+10% normal mouse serum, NMS) at 4°C overnight. Negative control sections were incubated with dilution buffer alone (PBS with 1% BSA and 10% NMS). All sections were washed and then incubated with biotinylated rabbit anti-rat secondary Ab (1:300 in dilution buffer; Dako Corporation, Carpinteria, CA) at 4°C for 2 h. The tissue sections were washed again in PBS, and incubated with Avidin-HRP conjugate (1:400 in dilution buffer; Dako Corporation) at 4°C for 2 h. SigmaFAST DAB tablet set (diaminobenzindine tetrachloride (0.7 mg/ml) and urea hydrogen peroxide (0.67 mg.ml); Sigma-Aldrich, St. Louis, MO) was employed to develop HRP staining followed by incubation for 10 min at room temperature. Sections were counterstained with hematoxylin (Sigma-Aldrich) and dehydrated in two changes of absolute ethanol, cleared in two changes of Safsolvent (Ajax Finechem), and mounted in Depex (BDH Laboratory Supplies, Toronto, ON, Canada). Video image analysis (VIA) software (Video Pro 32; Leading Edge, Blackwood, SA, Australia) was used to quantify the density of positive (DAB) staining in the uterine tissue sections. Prior to each session of data collection, the VIA system was calibrated using a standard field of tissue. The mean area of positive staining (% positivity) was quantified in 10 medium-power fields (x20 objective) from each horn in two non-serial sections of endometrial stroma. Data were expressed as a percentage calculated as (mean area of DAB stain/[mean area of hematoxylin + DAB stain] × 100).

### RNA analysis

RNA was prepared from uterine tissue and from various male organs as previously (Heng et al., 2012), using the Trizol reagent (Life Technologies, Mulgrave, Australia) and subsequently subjected to treatment with RNAse-free DNAse (TURBO DNA-*free*™; Life Technologies) according to the manufacturer's instructions. Because it proved difficult to process prostate tissue without incurring significant RNA degradation, RNA from CD1 and CD57BL/6 mouse prostate was also obtained from Amsbio (Abingdon, UK). cDNA was prepared from 3 μg total RNA per sample using oligo(dT) priming and Superscript II (Life Technologies) reverse transcriptase, again according to the manufacturer's instructions. Quantitative real-time PCR (qRT-PCR) was carried out using a Corbett Rotor-Gene 3000 (Qiagen, Melbourne, VIC, Australia) and the ExTaq Sybr-Green pre-mix (Takara, Shiga, Japan), with oligonucleotide primers and PCR conditions as listed in Supplementary Table 2. Primer pairs were designed using Primer 3 software to span at least one exon-exon boundary. Transcript levels were normalized against the ribosomal protein transcript S27a (*Rps27a*; Lee et al., 2002). Results were analyzed using Q-gene96 software (Muller et al., 2002). PCR products were validated by inspection of melt curves, as well as electrophoresis and sequencing of the final products for all genes. Additionally, RNA extracted in experiment two was subjected to Gene Signature Low Density Array RT-PCR analysis (LDAP; Life Technologies) using the Mouse Immune array (#4367786) exactly following the manufacturer's instructions, and employing an Applied Biosystems 7900HT analysis system. With this microfluidics system, 96 independent immune-related mouse gene transcripts could be analyzed with high fidelity from eight uterine RNA samples originating from *Rln*^−/−^ female mice mated with *Rln*^−/−^ males (*n* = 4) or *Rln*^+/+^ males (*n* = 4). Samples were selected on the basis of RNA integrity and quality, assessed by UV spectrophotometry (A260/A280 ratio > 2) and gel electrophoresis (28S/18S integrity) as well as by high consistency in the CT-values for housekeeping transcripts. Results were normalized against internal *Gapdh, Actb*, and *Rn18s* ribosomal RNA housekeeping targets.

### Analysis of uterine lavage

All uterine lavage samples were frozen at −80°C until analysis. For experiment 1, only CXCL1 (mouse KC) was assessed using a commercial ELISA kit (DY453; R&D Systems; Minneapolis, MN) exactly as per the manufacturer's instructions. For experiment 2, all samples were analyzed for cytokine and chemokine content using the Luminex multiplex system (Milliplex MAP kit, mouse cytokine/chemokine catalog #MPXMCYTO-70K; Merck-Millipore, Kilsyth, VIC, Australia) with a panel of 15 targets (CSF1, CSF2, CSF3, IL1A, IL1B, IL6, IL10, CXCL1, CXCL10, LIF, CCL2, CCL3, CCL4, CCL5, and TNFα). The manufacturer's protocol was followed precisely.

### Statistics

All data sets were analyzed by ANOVA followed by Neumann-Keuls *post-hoc* test or by Student's *t*-test to check individual comparisons. Because of marked skewness in the data, the Luminex cytokine data were log transformed prior to statistical analysis. *p* < 0.05 was considered significant.

## Results

### Expression of relaxin in the male reproductive tract and impact of relaxin gene deletion

**Figure 1:**
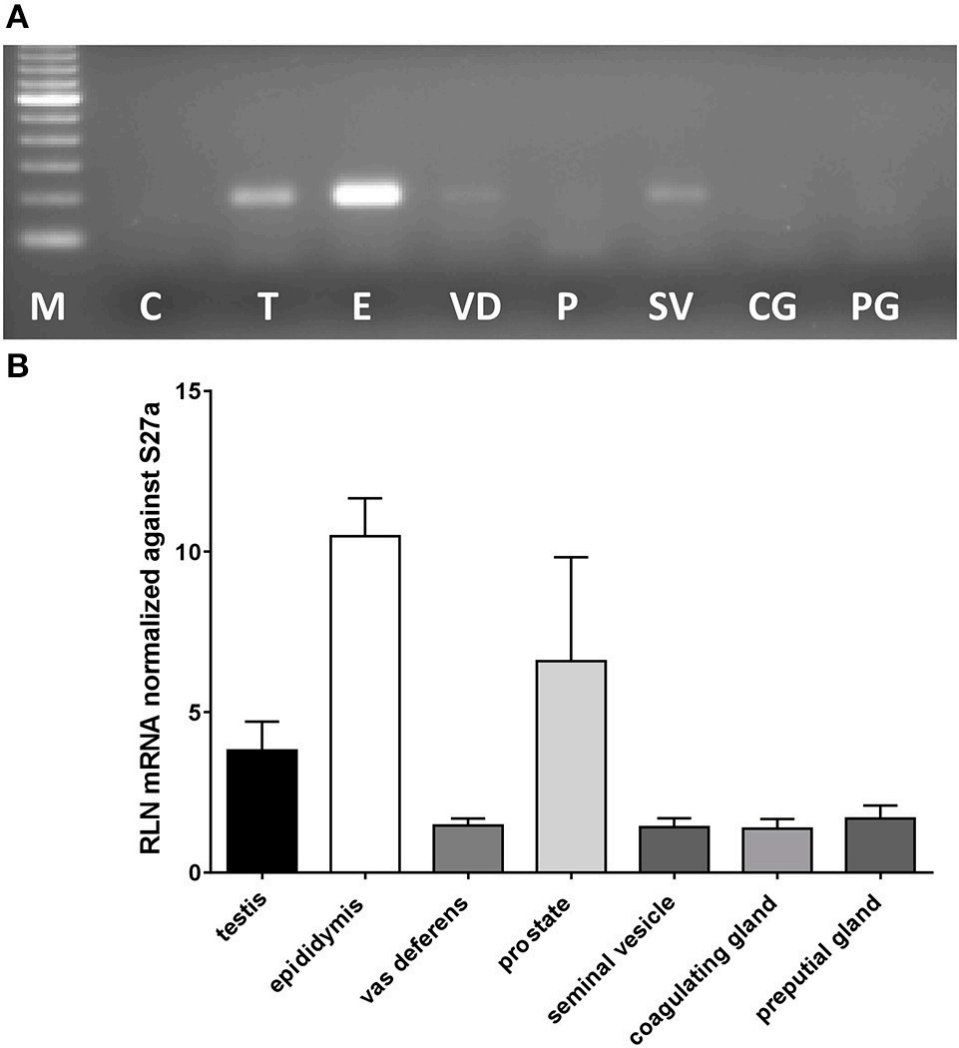
Real-time qRT-PCR quantification of Rln transcript levels in RNA extracted from testis (T), epididymis (E), vas deferens (VD), prostate (P), seminal vesicles (SV), coagulating glands (CG), and preputial glands (PG) from male wild type mice. Note that whereas most tissues derived from the DUK strain, prostate additionally derived from CD1 and CD57/Black 6 strains. **(A)** Example of PCR products from individual tissues following limited PCR cycles (this included prostate from DUK mice). **(B)** Relative transcript level normalized against Rps27a (mean ± SEM; *n* = 3–4 individuals per tissue).

In most mammals the likely source of seminal relaxin is probably the prostate and /or the seminal vesicles. In mice, based on their expression of relaxin mRNA, the main sources would appear to be in descending order: epididymis, prostate, testis, then seminal vesicles and vas deferens, with coagulating and preputial glands having only trace amounts of specific mRNA (Figure 1). Prostate mRNA proved to be variable with expression of *Rln* transcripts possibly depending on mouse strain. Our results are very similar to those presented by Ganesan et al. (2009), who also indicated low but variable expression of prostate relaxin mRNA for C57BL/6 mice. TGFß1, TGFß2, and TGFß3 are suggested to be amongst the major immunomodulatory components of seminal plasma (Robertson et al., 2002) and are also expressed in the organs of the male tract with TGFß1 and TGFß2 being quantitatively more important than TGFß3 (Robertson et al., 2002). In order to determine whether the lack of relaxin in male organs might be influencing the expression of TGFß and hence indirectly affecting the immunomodulatory properties of seminal plasma, TGFß gene expression was compared in the epididymides and testes, as the main sources of male relaxin, from relaxin knockout and sibling wild type mice (Supplementary Figure 1). For none of the TGFß subtypes was any difference noted.

### Effect of exogenous recombinant human relaxin on uterine CXCL1 synthesis *In vivo*

**Figure 2:**
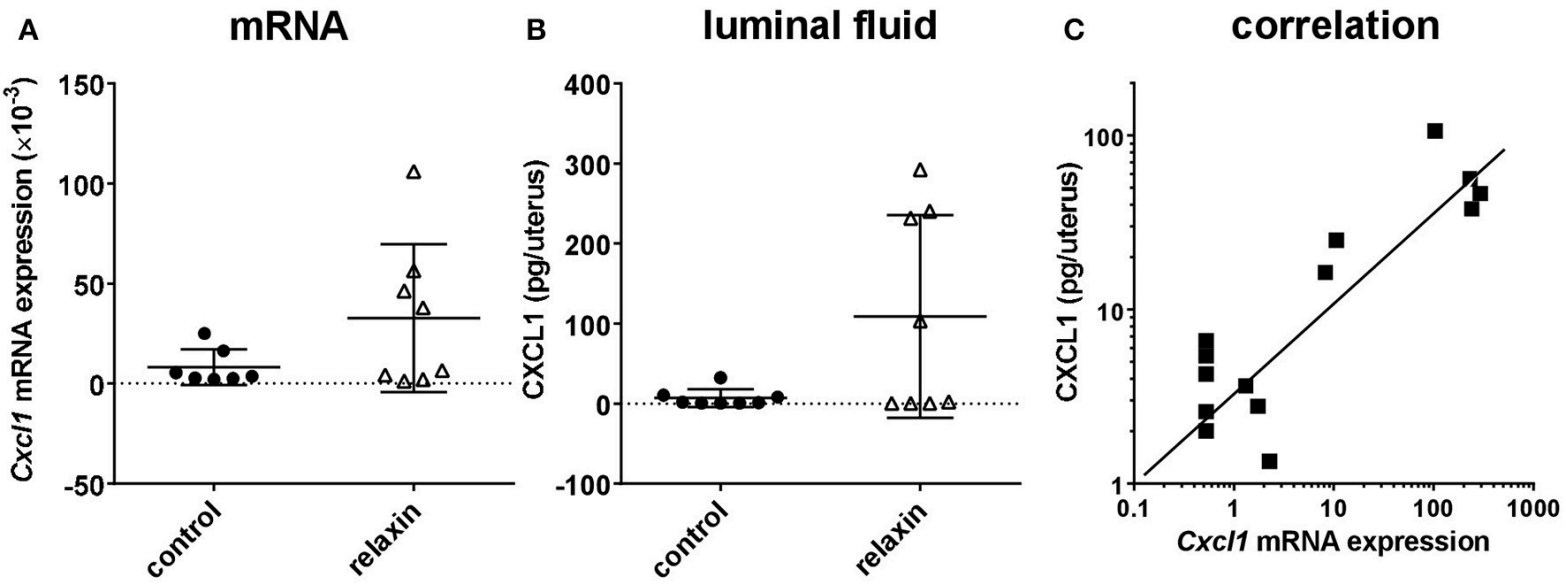
Effect of recombinant human relaxin (rhRLN) injected into the uterine horns at estrus on uterine *Cxcl1* mRNA expression assessed by qRT-PCR **(A)**, and CXCL1 secretion into uterine luminal fluid assessed by specific ELISA **(B)**, and the correlation between these parameters for individual mice **(C)**. Data are mean ± SEM (*n* = 7–8 per category). In panel **(C)**, symbols indicate data from mice both receiving or not rhRLN.

In the first experiment, exogenous rhRLN or vehicle was injected into the lumen of both left and right uterine horns in healthy virgin adult estrous *Rln*^+/+^ mice, i.e., mimicking the expected time of natural insemination. Ten hours later mice were sacrificed, uterine flushings collected and uterine tissue frozen in liquid nitrogen for later preparation of RNA for qRT-PCR analysis (left horns), or frozen in OCT medium for immunohistochemical evaluation (right horns). qRT-PCR analysis was carried out to quantify mRNA for *Cxcl1*, which encodes a typical pro-inflammatory cytokine, CXCL1 (mouse KC), which is known to be secreted from the mouse uterine epithelium (Robertson et al., 1998; Wood et al., 1999; Orsi et al., 2007). Moreover, this cytokine was selected for this pilot study as its functional equivalent in humans, IL-8, is secreted in response to relaxin by extraplacental chorionic cytotrophoblast (Bryant-Greenwood et al., 2009). In 50% of the mice *Cxcl1* mRNA appeared to be considerably altered by the relaxin treatment (Figure 2A), though some animals did not appear to respond. Evaluation of uterine flushings by cytokine-specific ELISA supports the gene expression results, with a similar increase in CXCL1 peptide secreted into the uterine lumen (Figure 2B). However, these differences were not statistically significant, largely due to some individual animals not responding to the treatment. In support of this interpretation, a strong correlation between *Cxcl1* mRNA and CXCL1 peptide from the same individuals is evident (Figure 2C). Immunohistochemical examination of uterine sections using two different antibodies to detect all leukocytes (CD45; TIB122) or neutrophils (RB6-8C5), showed no significant differences between treatment groups (Supplementary Figure 2D), implying that relaxin alone injected into the uterus did not lead to marked recruitment of immune cells. This did not differ even when comparing responders and non-responders (not shown). Similar results were obtained using qRT-PCR to evaluate the expression of *Ptprc* mRNA encoding CD45 (Supplementary Figure 2C).

### Effect of endogenous relaxin delivered by natural insemination on the mouse uterus *In vivo*

In the second set of experiments, we investigated the effect of genetic relaxin deficiency in either male semen and seminal fluid or the female reproductive tract. Wild-type female mice (*Rln*^+/+^), expressing ovarian relaxin, as well as *Rln*^−/−^ female mice were mated with either *Rln*^+/+^ or *Rln*^−/−^ males. These mating combinations provided the opportunity to evaluate the relative contributions of both seminal relaxin and endogenous ovarian relaxin in regulating the female post-mating cytokine response. Unlike the first experiment, analysis has made use of a more unbiased approach using LDAP cytokine mRNA arrays as well as cytokine/chemokine Luminex immunoarrays.

### Comparing Rln^−/−^ and Rln^+/+^ males on a Rln^−/−^ female background

The preliminary cytokine mRNA LDAP analysis was restricted to comparing gene transcripts which were either up- or down-regulated when uterine tissue was derived from *Rln*^−/−^ females that were mated with *Rln*^+/+^ compared with *Rln*^−/−^ males (Table 1). Only 14 of the 96 cytokine-related gene transcripts represented in these arrays were altered in their expression. The array data suggested that several cytokines and chemokines involved in regulating the inflammatory response may be expressed at higher levels in *Rln*^−/−^ females mated with *Rln*^−/−^ males compared with *Rln*^+/+^ males (including *Ccl2, Csf1, Csf3, Cxcl10*, and *Il6*, all *p* < 0.05). This initial analysis was used to identify potential candidates for in-depth qRT-PCR measurement.

**Table 1:** Gene transcripts significantly altered in *Rln*^−/−^ females when mated with *Rln*^+/+^ compared to *Rln*^−/−^ males using the mouse cytokine LDAP array.

**Gene**	**Names and features**	**Up/down**	**Fold change**	**Significance**
*Agtr2*	Angiotensin II receptor, type 2	Up	2.2	*p* = 0.01
*Bcl2*	Anti-apoptotic regulator	Up	1.6	*p* = 0.04
*Ccl2*	MCP1	Down	0.1	*p* = 0.01
*Ccl3*	MIP-1α	(Down)^1^	0.3	*p* = 0.07
*Cd80*	T cell modulator	Down	0.4	*p* = 0.04
*Csf1*	M-CSF	Down	0.6	*p* = 0.01
*Csf3*	G-CSF	Down	0.1	*p* = 0.01
*Cxcl10*	IP-10	Down	0.2	*p* = 0.02
*Ece1*	Endothelin-converting enzyme 1	Down	0.6	*p* = 0.02
*Il18*	Interleukin 18	(Up)	1.7	*p* = 0.07
*Il6*	Interleukin 6	Down	0.1	*p* = 0.03
*Ptgs2*	Cyclooxygenase type 2	(Down)	0.2	*p* = 0.10
*Ptprc*	CD45	Up	1.6	*p* = 0.01
*Smad3*	Downstream TGFß signaling	Up	1.5	*p* = 0.02

1 *parentheses indicate that the fold-change was significant at the p < 0.1 but not the p < 0.05 level*.

**Figure 3:**
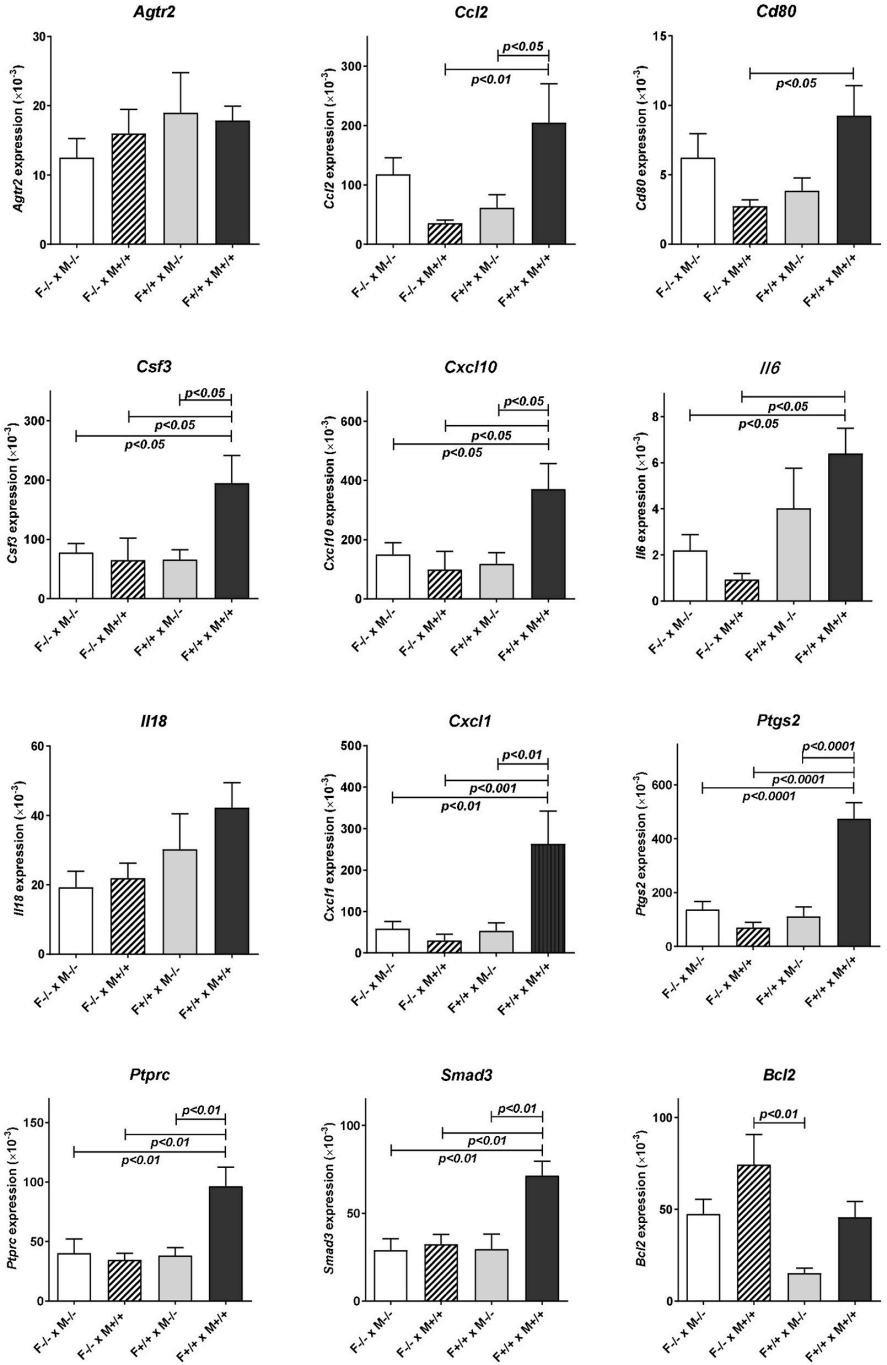
Real-time qRT-PCR quantification of specific transcript levels in RNA extracted from the individual uteri of different mating combinations utilizing Rln^+/+^ and Rln^−/−^ male and female mice, as indicated. Data are mean ± SEM. *p*-values indicating effect of treatment group on cytokine mRNA concentration are shown.

**Figure 4:**
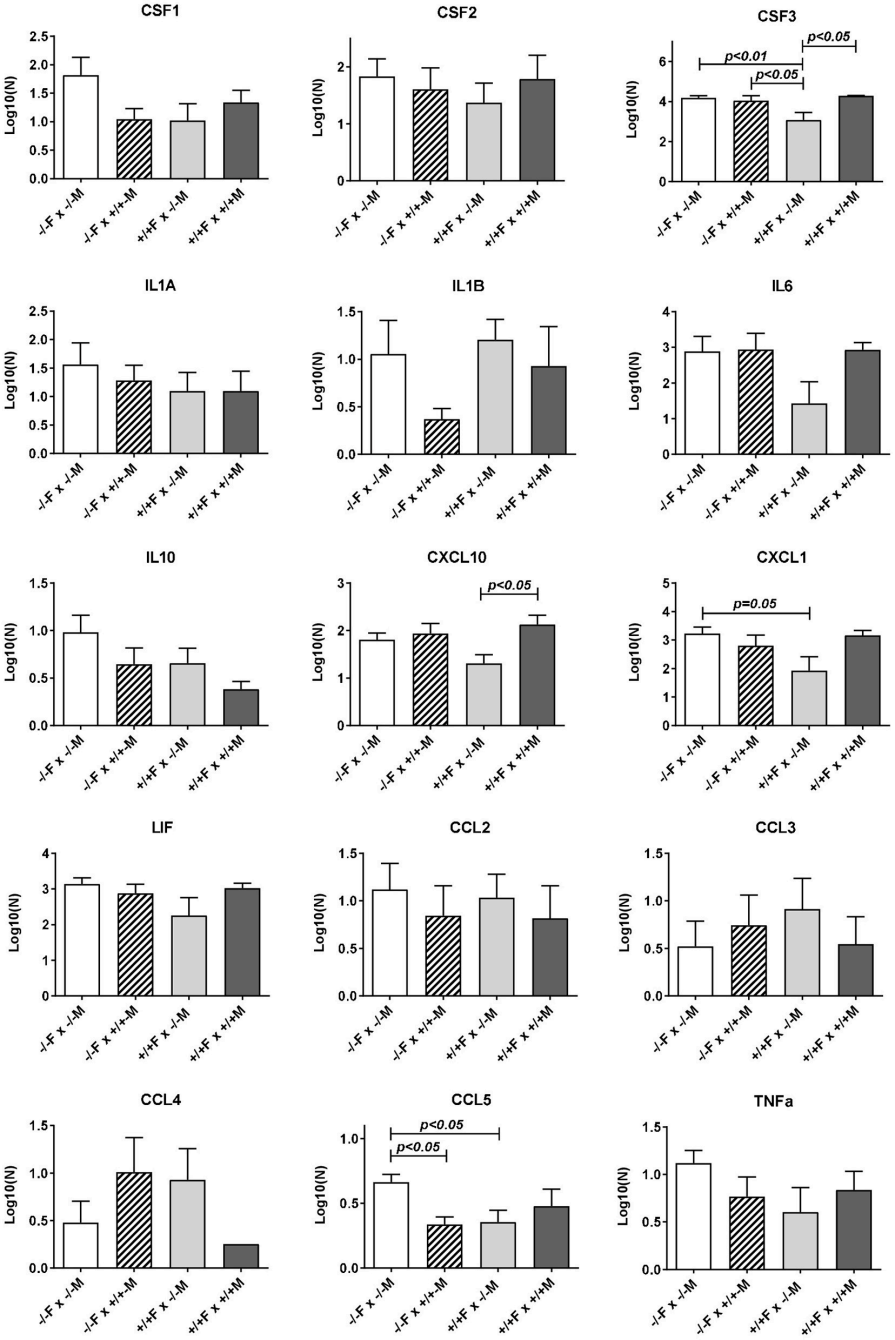
Luminex multiplex analysis of individual cytokines and chemokines present in uterine lavage fluid derived from different mating combinations utilizing Rln^+/+^ and Rln^−/−^ male and female mice, as indicated. Data were log transformed prior to analysis, and are given as mean ± SEM. *p*-values indicating effect of treatment group on cytokine content are shown.

Subsequent qRT-PCR analysis of these apparently regulated genes (Figure 3), partly confirmed the LDAP array results for *Ccl2, Cd80, IL6*, and *Ptgs2*, in that these four gene transcripts also showed a similar trend toward reduction caused by mating the *Rln*^−/−^ females with *Rln*^+/+^ males, compared to *Rln*^−/−^ males, though this was just not statistically significant. Measurement of cytokines in the uterine lavage from these matings showed a significant reduction on mating to *Rln* ^+/+^ males only for CCL5, though similar trends were apparent also for CSF1 and IL1b (Figure 4).

### Comparing Rln^−/−^ and Rln^+/+^ males on a Rln^+/+^ female background

Uterine RNA was recovered from *Rln*^+/+^ females mated with both *Rln*^−/−^ and *Rln*^+/+^ males and analyzed by qRT-PCR, to evaluate whether similar changes to those above were observed when female relaxin was intact (Figure 3). In this comparison, the absence of male relaxin caused a different shift in the pattern of response, with substantially reduced uterine expression of several cytokine and chemokine genes including *Ccl2, Csf3, Cxcl10*, and *Cxcl1* after *Rln*^+/+^ females were mated with *Rln*^−/−^ compared with *Rln*^+/+^ males. Additionally, *Ptprc*, encoding the leukocyte common antigen CD45, *Cd80, Ptgs2, Smad3*, and *Bcl2* were similarly all down-regulated in the absence of male relaxin.

Uterine flushings from the same mating combinations (*Rln*^+/+^ females vs. *Rln*^−/−^ or *Rln* ^+/+^ males; Figure 4) were analyzed by the Luminex cytokine system. Whilst for most products individual variation was too high to yield statistical significance, for both CSF3 and CXCL10, there was a significant (*p* < *0.05*) reduction upon mating with *Rln*^−/−^ males, reflecting that seen at the mRNA level, as also a strong trend to reduction for CXCL1 (*p* = 0.08).

### Comparing Rln^−/−^ and Rln^+/+^ females on a Rln^+/+^ male background

Comparison of uterine gene expression in *Rln*^−/−^ and *Rln*^+/+^ females after mating with *Rln*^+/+^ males also showed substantial effects for several gene transcripts (Figure 3), indicating that the presence or absence of relaxin in the female mice was not without influence. In *Rln*^−/−^ females, a substantial reduction in expression of cytokine and chemokine genes *Ccl2, Csf3, Cxcl1, Cxcl10*, and *Il6* was evident (Figure 3). This was accompanied by significantly diminished expression of *Cd80, Ptprc (Cd45), Ptgs2*, and *Smad3* (Figure 3). No significant differences for this mating combination were revealed at the level of cytokine peptides (Figure 4).

### Other mating combinations

As might be anticipated, when complete absence of relaxin in both males and females is compared to full wild type matings, then significant decreases in uterine mRNA in the *Rln*^−/−^ females are observed for *Csf3, Cxcl10, IL6, Cxcl1, Ptgs2, Ptprc* (*Cd45*), and *Smad3* (Figure 3), i.e., essentially similar to the effects seen comparing female *Rln*^−/−^ with female *Rln*^+/+^ both mated with *Rln*^+/+^ males. Also as for this mating combination, no effects were evident at the level of peptides in the uterine washings (Figure 4). Only for *Bcl2* mRNA was there any significant difference at the transcript level for the reciprocal matings female *Rln*^−/−^ with male *Rln*^+/+^ vs. female *Rln*^+/+^ with male *Rln* ^−/−^ (Figure 3), and at the level of peptides in the uterine lavage fluid, only CSF3 indicated a significant difference (Figure 4).

As controls, the transcript levels for relaxin itself as well as for its specific receptor RXFP1 were also measured. Other than the absence of relaxin in the uteri of *Rln*^−/−^ mice, no differences in expression were evident (Supplementary Figures 2E,F).

## Discussion

It is not possible directly to assess relaxin in mouse seminal fluid since electroejaculatory techniques are not well developed for this species, and the usual method of using uterine lavage following mating is logically prohibited since relaxin may be secreted or metabolized within the female tract. Therefore, we made an exhaustive RT-PCR analysis of those male organs likely to be contributing most to seminal fluid. The mammalian ejaculate is not uniform but rather comprises different secretory elements delivered sequentially to achieve different roles. The initial ejaculate comprises largely epididymal fluid containing sperm and some testicular contribution, together with some secretion from the prostate and preputial glands (Rodríguez-Martínez et al., 2011). This is followed by increasing amounts of prostatic and seminal vesicle fluid, which together alter the physical properties of the ejaculate. Last in rodents are the secretions of the coagulating gland. The observation that relaxin is a product mostly of the epididymis, followed by the prostate and testis in mice, is therefore fully compatible with the view that the initial ejaculate may be the most important contributor to the immunomodulatory dialog between seminal plasma and the uterus. Whilst, it is proposed that the TGFß family of peptides may be amongst the most important components of seminal plasma in the context of immune adjustment of the uterus (Esche et al., 2005), we have shown here that none of the TGFß subtype transcripts appear to be altered in the testis and epididymis, representative of the main relevant relaxin-producing organs of the male system in homozygous relaxin knockout mice. Since relaxin in the male is rather a paracrine/autocrine than an endocrine acting hormone, it is unlikely that TGFβ will be altered in those tissues not expressing relaxin. Taken together these findings strongly support the view that the effects observed in the present studies are due to relaxin, probably of epididymal and/or prostatic origin, present in murine seminal fluid at ejaculation, although a role for spermatozoa as passive carriers of relaxin cannot be excluded.

The principal aim of this study was to define the contribution of male relaxin in eliciting expression of cytokines and chemokines in the uterine post-mating cytokine response. Using two complimentary strategies involving either administration of exogenous relaxin or analysis of the effects of genetic relaxin deficiency, we show herein that male relaxin is a contributing factor in regulating uterine synthesis particularly of the cytokines CCL2, CSF3, CXCL1, and CXCL10 during the 10 h period following mating, and also of the downstream effectors PTGS2, and SMAD3. These changes are also associated with an increase due to male relaxin of uterine mRNA for the pan-leukocyte antigen PTPRC (CD45), at least in the cross-mating experiment, implying that there may indeed be a net increase in uterine leukocytes following mating with *Rln*^+/+^ males. The apparent difference for CD45 between the LDAP results (Table 1; up-regulated by relaxin on a *Rln*^−/−^ female background) and the qRT-PCR results (Figure 3; no change for the same mating combination) may be due to the larger number of RNA samples used in the latter analysis.

CSF3 and CXCL1 are identified as regulators of neutrophil recruitment and activation, while CXCL10 targets monocyte macrophages, dendritic cells, and lymphocytes (Esche et al., 2005). Neutrophils, macrophages, and dendritic cells are prevalent amongst the leukocytes infiltrating the uterine stroma and lumen after semen deposition (McMaster et al., 1992; Robertson et al., 1998), and these cells have key roles in the immune adaptations as well as structural changes required for uterine receptivity at implantation (Esche et al., 2005; Plaks et al., 2008; Moldenhauer et al., 2009).

In the first approach used, recombinant human relaxin was injected directly into the uteri of estrous wild-type female mice, at a dose presenting moderately supraphysiological concentrations of relaxin to the luminal surface of the endometrium. Ten hours later, animals were sacrificed, uteri flushed, and tissues collected for qRT-PCR and immunohistochemistry. After this time period in these cycling virgin mice only persistent, non-acute effects of the administered relaxin are likely to be observed. It is therefore not unexpected that some animals might indicate no effect at this time-point. CXCL1 (mouse KC) was chosen as indicator, since it has been previously shown to be a reliable marker of specific uterine epithelial secretion in the mouse (Orsi et al., 2007) and is known to be elevated after mating (Wood et al., 1999). Moreover, CXCL1 is considered to be a functional equivalent to IL8 in the human female tract, and IL8 expression has been shown to be stimulated by relaxin both in human cytotrophoblast cells and decidual macrophages (Horton et al., 2011, 2012), as well as to be induced by seminal fluid in the human cervix after coitus (Sharkey et al., 2012b). Although, the large differences between relaxin stimulation and vehicle, both for mRNA and peptide expression in this experiment, failed to reach significance due to apparent non-responder mice, the significant correlation (*p* < 0.001) between peptide and mRNA expression strongly implies that the variance in this model is not of a technical nature, but is due to the biological variability in mouse responsiveness. Histological examination indicated no obvious morphological correlates possibly indicative of infection. Moreover, in this experiment, no differences were noted in terms of leukocyte recruitment to the uterus as assessed by immunohistochemistry and qRT-PCR (Supplementary Figure 2), though such methods may not be sufficiently sensitive at this time-point to identify real changes in leukocyte numbers.

Following this preliminary study which indicated that pure exogenous relaxin introduced into the uterine lumen at estrous can have an effect on the uterine cytokine response, the second experiment was designed to test the effect of seminal relaxin in the context of natural mating. It is important to note here that the mRNA measured by the LDAP array and by individual qRT-PCR reflected all components of the uterus, including myometrial, epithelial, and stromal cells, as well as resident leukocytes. In contrast, the proteins detected in the uterine lavage are presumably secreted by the uterine epithelium and possibly resident leukocytes, which in the period after mating are abundant in the endometrial tissue subjacent to the epithelial surface, as well as within the uterine lumen (McMaster et al., 1992; Robertson et al., 1998). Thus, they represent a subset of the total proteome of the endometrium and also may be lacking components secreted acutely and subsequently degraded or resorbed.

In addition to the effects of male relaxin discussed above, significant effects of endogenous female relaxin were also evident in experiments comparing *Rln*^−/−^ females with *Rln*^+/+^ females. Mating with wild type (*Rln*^+/+^) males resulted in a significantly diminished uterine expression in the absence of endogenous relaxin of transcripts for *Ccl2, Cd80, Csf3, Cxcl1, Cxcl10, Il6, Ptgs2, Ptprc (CD45)*, and *Smad3*. This suggests that pre-exposure of the female to endogenous relaxin in some way potentiates the female response to seminal fluid introduced during mating. This was not reflected in any change in secreted cytokines at the peptide level (the remaining gene products were not evaluated). However, since the experimental approach evaluated only proteins secreted at the apical surface of the epithelium or by cells within the lumen, but not basally secreted proteins or stromal cell products, the possibility that more detailed analysis would reveal protein differences cannot be discounted.

The effects of female relaxin deficiency were less pronounced when responses to mating with *Rln*^−/−^ males were evaluated. qRT-PCR analysis showed no significant effect of the presence or not of female relaxin in this context. At the level of uterine lavage, however, differences were seen for CSF3, CXCL1, and CCL5, with the presence of relaxin in the female leading to a diminution in these peptides. One interpretation of this data, taken together with data from matings with *Rln*^+/+^ males, is that the potentiating effect of relaxin on female tract responsiveness particularly affects the response to the relaxin component of the seminal fluid signaling activity. Whilst, it could be expected that chronic relaxin absence in null mutant females might lead to a change in the expression of the relaxin receptor, RXFP1, or otherwise alter the relaxin signaling cascade, no differences in *Rxfp1* mRNA expression were observed (Supplementary Figure 2), as had been previously reported for *Rln*^−/−^ mice (Siebel et al., 2003).

When the effects at the mRNA level of male *Rln*^−/−^ mice are compared with those of male *Rln*^+/+^ mice after mating to *Rln*^−/−^ females, as measured by individual qRT-PCR, there is no significant change for any transcript when measured using qRT-PCR. This thus differs from the LDAP array analysis (Table 1), where altogether 14 transcripts appeared to be significantly either up- or down-regulated. Whereas, the LDAP arrays were limited to four individuals from each mating combination with the *Rln*^−/−^ females, the subsequent individual qRT-PCR analyses included all mating combinations with between five and nine uterine samples, each from a separate female, included in each combination. Thus, the latter analysis inherently comprises more potential for biological variation. At the peptide level for the mating combinations involving *Rln*^−/−^ females, there is also only a significant down-regulation due to male relaxin observed for CCL5. Thus, in the absence of any female relaxin, seminal relaxin either has no significant effect or acts to down-regulate the pro-inflammatory cytokine network.

This is quite different where the female mice express endogenous relaxin. On the *Rln*^+/+^ female background, mating with *Rln*^+/+^ males, as opposed to *Rln*^−/−^ males, leads to the specific up-regulation of several transcripts and/or secreted peptides representing the pro-inflammatory cytokine network (specifically *Ccl2, Cd80, Csf3, Cxcl10, Cxcl1, Ptgs2, Ptprc (Cd45), Smad3, Bcl2*). No gene products are significantly down-regulated in this mating combination. Importantly, for *Csf3* and *Cxcl10* gene products the up-regulation is significant at both mRNA and secreted peptide levels, providing confidence in the interpretation that these factors are indeed regulated by seminal relaxin.

There is considerable evidence to show that one of the main components of mouse seminal plasma, TGFß, has a significant influence on the immune competence of the inseminated uterus (Rodríguez-Martínez et al., 2011). There is no evidence, however, to suggest that TGFß production or seminal vesicle function is impaired in the *Rln*^−/−^ males (Samuel et al., 2003; see also Supplementary Figure 1), nor does there appear to be any impact of the mutation on breeding performance parameters (Zhao et al., 1999; Glynn and Ivell, unpublished). Thus, it is interesting to note that amongst the cytokines specifically affected by the male relaxin gene knockout, the specific downstream TGFß effectors SMAD3 and PTPRC (CD45) are up-regulated, as is also the inducible prostaglandin synthase, PTGS2. The implicated cytokines have also recently been shown to be part of the TLR4 signaling cascade in the mouse uterus responsive to seminal fluid (Schjenken et al., 2015). It therefore seems likely that male relaxin is working additively with TGFß, to induce immune tolerance in the female in preparation for implantation. This is also supported by the specific up-regulation by mating with the *Rln*^+/+^ males of *Cxcl10* and the tolerogenic dendritic cell marker *Cd80*.

It has been shown that seminal plasma *per-se*, and in particular the TGFß component of this, are important for the recruitment of leukocytes to the uterus and their involvement in the development of immune tolerance to potential paternal alloantigens expressed by the fetus (Robertson et al., 2006; Robertson, 2007). Some of these experiments made use of relatively gross manipulations of seminal plasma, for example, mating with animals which had had their seminal vesicles surgically removed (Johansson et al., 2004). In the experiment described here all of the components of seminal plasma, except for seminal relaxin, are unchanged. Given the known redundancy amongst the broad panel of chemokines induced in the uterus by seminal fluid (McMaster et al., 1992; Robertson et al., 1998), it is possible that the moderate changes in chemokine and cytokine levels reported in this study might be insufficient to cause major alterations in the relative abundance of different recruited leukocytes in the endometrium. Thus, in the first experiment, where recombinant relaxin was introduced into the uteri of wild-type female mice, we failed to detect any impact on recruited leukocytes, using immunohistochemistry or qRT-PCR. Nevertheless, in the second study using different matings of *Rln*^−/−^ mice, uterine mRNA for the pan-leukocyte antigen CD45, as also the dendritic cell marker CD80, were indeed significantly altered in direct association with the changes in specific cytokine transcripts. Future studies to evaluate the precise phenotypes and functional competence of leukocytes in activities including antigen presentation and fate determination of activated T cells would be warranted. It is relevant that previous studies in relaxin-deficient mice have not included analysis of allogeneic matings, where fetal-maternal MHC disparity might reveal a more profound role in maternal immune regulation than is suggested by existing reports.

One of the more interesting findings was the observation that the effect of seminal relaxin was markedly different on a female *Rln*^−/−^ compared to a female *Rln*^+/+^ background. The relaxin contributed by the female may act in some way to “prime” or sensitize the immune response within the uterus. This female effect may also be simply additive or synergistic in regard to effects of relaxin in the female tract, whereby the uterine lumen could be considered as an independent compartment separated from the endometrium by the tight junctions of the luminal epithelium (Robertson et al., 1997; Wira et al., 2005). Female relaxin, which at the time of insemination in rodents is secreted both by granulosa cells of pre-ovulatory follicles as well as by the corpus luteum, most likely uses a vascular route to target receptors on endometrial stromal and vascular cells, as well as the myometrium, and possibly also the baso-lateral compartment of the luminal epithelium. In female reproductive physiology, relaxin has been shown to influence particularly the stromal cells to induce decidualization, and later to support angiogenesis and vasodilation (Ivell and Einspanier, 2002; Goldsmith et al., 2004), besides synergizing with estrogen to cause the typical uterine edema observed at implantation (Pillai et al., 1999). Thus, at estrus ovarian relaxin is likely already activating specific receptors within the uterus, at the time when seminal relaxin is added by insemination.

Finally, there is the question of whether, besides activating cytokines of epithelial origin, relaxin is also able to stimulate immune cells directly. It has been long established that relaxin is able to stimulate PKA-dependent pathways within human monocytes (Bartsch et al., 2001), directly inducing effects on adhesion and migration that might facilitate monocyte/macrophage recruitment in an inflammatory response (Figueiredo et al., 2006). Moreover, relaxin has been shown *in vitro* to promote the formation of Th1 cells, which produce the anti-inflammatory cytokine IFNγ, rather than Th2 cells (Piccinni et al., 1999). However, a skewing in favor of Th1 cells would seem at odds with emerging data that Treg cells are the predominant phenotype elicited by seminal fluid exposure (Robertson et al., 2009), so future experiments in relaxin null mutant mice are required to investigate this.

In conclusion, these studies in mice show for the first time that there is an important role for relaxin of male origin to act in concert with other proinflammatory agents in seminal fluid, such as TGFß, in order to initiate the cytokine response underpinning maternal immune adaptation for embryo implantation and development. Furthermore, as a corollary, female relaxin may act to sensitize the uterus to such effects, since both male and female relaxin appear to be required for maximal cytokine expression and effect. However, the fact that *Rln*^−/−^ mice are essentially fertile shows that there is a degree of redundancy in such informational processing. These findings are of necessity preliminary and will require more intensive study in the future, making use of other time-points and possibly including animals with inactivated relaxin receptors.

## Author contributions

RI was responsible for the overall conception and management of the project, and contributed significantly to writing the manuscript. DG, KH, DR, DS, and RA all contributed to the experimental work and interpretation of results. RA and SR shared responsibility with RI in the writing and overall interpretation of results.

### Conflict of interest statement

The authors declare that the research was conducted in the absence of any commercial or financial relationships that could be construed as a potential conflict of interest.

## References

[B1] Almeida I. L. Durfey C. L. Gastal G. D. Devos-Burnett D. Willard S. T. Ryan P. L.. (2016). Relaxin and its receptors in mature canine spermatozoa. Reprod. Fertil. Dev. 29:175. 10.1071/RDv29n1Ab13428278931

[B2] Anand-Ivell R. Ivell R. (2014). Regulation of the reproductive cycle and early pregnancy by relaxin family peptides. Mol. Cell. Endocrinol. 382, 472–479. 10.1016/j.mce.2013.08.010 23994019

[B3] Anand-Ivell R. Heng K. Bartsch O. Ivell R. (2007). Relaxin signalling in THP-1 cells uses a novel phosphotyrosine-dependent pathway. Mol. Cell. Endocrinol. 272, 1–13. 10.1016/j.mce.2007.04.001 17509748

[B4] Bartsch O. Bartlick B. Ivell R. (2001). Relaxin signalling links tyrosine phosphorylation to phosphodiesterase and adenylyl cyclase activity. Mol. Hum. Reprod. 7, 799–809. 10.1093/molehr/7.9.799 11517286

[B5] Bartsch O. Bartlick B. Ivell R. (2004). Phosphodiesterase 4 inhibition synergizes with relaxin signalling to promote decidualization of human endometrial stromal cells. J. Clin. Endocrinol. Metab. 89, 324–334. 10.1210/jc.2003-030498 14715868

[B6] Bathgate R. A. Ivell R. Sanborn B. M. Sherwood O. D. Summers R. J. (2006). International Union of Pharmacology LVII: recommendations for the nomenclature of receptors for relaxin family peptides. Pharmacol. Rev. 58, 7–31. 10.1124/pr.58.1.9 16507880

[B7] Brannström M. MacLennan A. H. (1993). Relaxin induces ovulations in the *in-vitro* perfused rat ovary. Hum. Reprod. 8, 1011–1014. 10.1093/oxfordjournals.humrep.a138184 8408479

[B8] Bromfield J. J. (2014). Seminal fluid and reproduction: much more than previously thought. J. Assist. Reprod. Genet. 31, 627–636. 10.1007/s10815-014-0243-y 24830788 PMC4048384

[B9] Bryant-Greenwood G. D. Yamamoto S. Y. Sadowsky D. W. Gravett M. G. Novy M. J. (2009). Relaxin stimulates interleukin-6 and interleukin-8 secretion from the extraplacental chorionic cytotrophoblast. Placenta 30, 599–606. 10.1016/j.placenta.2009.04.009 19467703

[B10] Carrell D. T. Petersen C. M. Urry R. L. (1995). The binding of recombinant human relaxin to human spermatozoa. Endocr. Res. 21, 697–707. 10.1080/07435809509030484 7588437

[B11] Conrad K. P. (2011). Maternal vasodilation in pregnancy: the emerging role of relaxin. Am. J. Physiol. Regul. Integr. Comp. Physiol. 301, R267–R275. 10.1152/ajpregu.00156.2011 21613576 PMC3154715

[B12] Downing S. J. Sherwood O. D. (1985). The physiological role of relaxin in the pregnant rat. II. The influence of relaxin on uterine contractile activity. Endocrinology 116, 1206–1214. 10.1210/endo-116-3-1206 3971904

[B13] Einspanier A. Lieder K. Husen B. Ebert K. Lier S. Einspanier R.. (2009). Relaxin supports implantation and early pregnancy in the marmoset monkey. Ann. N. Y. Acad. Sci. 1160, 140–146. 10.1111/j.1749-6632.2009.03947.x 19416176

[B14] Esche C. Stellato C. Beck L. A. (2005). Chemokines: key players in innate and adaptive immunity. J. Invest. Dermatol. 125, 615–628. 10.1111/j.0022-202X.2005.23841.x 16185259

[B15] Ferlin A. Menegazzo M. Gianesello L. Selice R. Foresta C. (2012). Effect of relaxin on human sperm functions. J. Androl. 33, 474–482. 10.2164/jandrol.110.012625 21903973

[B16] Feugang J. M. Greene J. M. Sanchez-Rodriguez H. L. Stokes J. V. Crenshaw M. A. Willard S. T. (2015). Profiling of relaxin and its receptor proteins in boar reproductive tissues and spermatozoa. Reprod. Biol. Endocrinol. 1:46 10.1186/s12958-015-0043-y PMC4445784 25990010

[B17] Figueiredo K. A. Mui A. L. Nelson C. C. Cox M. E. (2006). Relaxin stimulates leukocyte adhesion and migration through a relaxin receptor LGR7-dependent mechanism. J. Biol. Chem. 281, 3030–3039. 10.1074/jbc.M506665200 16303766

[B18] Ganesan A. Klonisch T. McGuane J. T. Feng S. Agoulnik A. I. Parry L. J. (2009). Normal prostate morphology in relaxin-mutant mice. Reprod. Fertil. Dev. 21, 440–450. 10.1071/RD08133 19261221

[B19] Goldsmith L. T. Weiss G. Palejwala S. Plant T. M. Wojtczuk A. Lambert W. C.. (2004). Relaxin regulation of endometrial structure and function in the rhesus monkey. Proc. Natl. Acad. Sci. U.S.A. 101, 4685–4689. 10.1073/pnas.0400776101 15070778 PMC384807

[B20] Gunnersen J. M. Crawford R. J. Tregear G. W. (1995). Expression of the relaxin gene in rat tissues. Mol. Cell. Endocrinol. 110, 55–64. 10.1016/0303-7207(95)03516-A 7545623

[B21] Heng K. Anand-Ivell R. Teerds K. Ivell R. (2012). The endocrine disruptors dibutyl phthalate (DBP) and diethylstilbestrol (DES) influence Leydig cell regeneration following ethane dimethane sulfonate (EDS) treatment of adult male rats. Int. J. Androl. 35, 353–363. 10.1111/j.1365-2605.2011.01231.x 22150342

[B22] Horton J. S. Yamamoto S. Y. Bryant-Greenwood G. D. (2011). Relaxin modulates proinflammatory cytokine secretion from human decidual macrophages. Biol. Reprod. 85, 788–797. 10.1095/biolreprod.110.089201 21734258 PMC4480428

[B23] Horton J. S. Yamamoto S. Y. Bryant-Greenwood G. D. (2012). Relaxin augments the inflammatory IL6 response in the choriodecidua. Placenta 33, 399–407. 10.1016/j.placenta.2012.02.002 22386961 PMC3319264

[B24] Ivell R. Einspanier A. (2002). Relaxin peptides are new global players. Trends Endocrinol. Metab. 13, 343–348. 10.1016/S1043-2760(02)00664-1 12217491

[B25] Ivell R. Kotula-Balak M. Glynn D. Heng K. Anand-Ivell R. (2011). Relaxin family peptides in the male reproductive system – a critical appraisal. Mol. Hum. Reprod. 17, 71–84. 10.1093/molehr/gaq086 20952422

[B26] Johansson M. Bromfield J. J. Jasper M. J. Robertson S. A. (2004). Semen activates the female immune response during early pregnancy in mice. Immunology 112, 290–300. 10.1111/j.1365-2567.2004.01876.x 15147572 PMC1782486

[B27] Kachkache M. Acker G. M. Chaouat G. Noun A. Garabedian M. (1991). Hormonal and local factors control the immunohistochemical distribution of immunocytes in the rat uterus before conceptus implantation; effects of ovariectomy, fallopian tube section, and injection. Biol. Reprod. 45, 860–868. 10.1095/biolreprod45.6.860 1805988

[B28] Kohsaka T. Takahara H. Sasada H. Kawarasaki T. Bamba K. Masaki J.. (1992). Evidence for immunoreactive relaxin in boar seminal vesicles using combined light and electron microscope immunocytochemistry. J. Reprod. Fertil. 95, 397–408. 10.1530/jrf.0.0950397 1517997

[B29] Laird P. W. Zijderveld A. Linders K. Rudnicki M. A. Jaenisch R. Berns A. (1991). Simplified mammalian DNA isolation procedure. Nucleic Acids Res. 19:4293. 10.1093/nar/19.15.4293 1870982 PMC328579

[B30] Lee P. D. Sladek R. Greenwood C. M. Hudson T. J. (2002). Control genes and variability: absence of ubiquitous reference transcripts in diverse mammalian expression studies. Genome Res. 12, 292–297. 10.1101/gr.217802 11827948 PMC155273

[B31] MacLennan A. H. Grant P. Ness D. Down A. (1986). Effect of porcine relaxin and progesterone on rat, pig and human myometrial activity *in vitro*. J. Reprod. Med. 31, 43–49. 3950882

[B32] McMaster M. T. Newton R. C. Dey S. K. Andrews G. K. (1992). Activation and distribution of inflammatory cells in the mouse uterus during the preimplantation period. J. Immunol. 148, 1699–1705. 1541814

[B33] Miah A. G. Tareq K. M. A. Hamano K. Kohsaka T. Tsujii H. (2006). Effect of relaxin on acrosome reaction and utilization of glucose in boar spermatozoa. J. Reprod. Dev. 52, 773–779. 10.1262/jrd.18037 16926527

[B34] Millar L. K. Reiny R. Yamamoto S. Y. Okazaki K. Webster L. Bryant-Greenwood G. D. (2003). Relaxin causes proliferation of human amniotic epithelium by stimulation of insulin-like growth factor II. Am. J. Obstet. Gynecol. 188, 234–241. 10.1067/mob.2003.80 12548223

[B35] Moldenhauer L. M. Diener K. R. Thring D. M. Brown M. P. Hayball J. D. Robertson S. A. (2009). Cross-presentation of male seminal fluid antigens elicits T cell activation to initiate the female immune response to pregnancy. J. Immunol. 182, 8080–8093. 10.4049/jimmunol.0804018 19494334

[B36] Muller P. Y. Janovjak H. Miserez A. R. Dobbieet Z. (2002). Processing of gene expression data generated by quantitative real-time RT-PCR. Biotechniques 32, 1372–1379. 12074169

[B37] Orsi N. M. Ekbote U. V. Walker J. J. Gopichandran N. (2007). Uterine and serum cytokine arrays in the mouse during estrus. Anim. Reprod. Sci. 100, 301–310. 10.1016/j.anireprosci.2006.08.016 16963201

[B38] Parry L. J. Vodstrcil L. A. (2007). Relaxin physiology in the female reproductive tract during pregnancy. Adv. Exp. Med. Biol. 612, 34–48. 10.1007/978-0-387-74672-2_4 18161480

[B39] Parsell D. A. Mak J. Y. Amento E. P. Unemori E. N. (1996). Relaxin binds to and elicits a response from cells of the human monocytic cell line, THP-1. J. Biol. Chem. 271, 27936–27941. 10.1074/jbc.271.44.27936 8910395

[B40] Piccinni M. P. Bani D. Beloni L. Manuelli C. Mavilla C. Vocioni F.. (1999). Relaxin favors the development of activated human T cells into Th1-like effectors. Eur. J. Immunol. 29, 2241–2247. 10.1002/(SICI)1521-4141(199907)29:07<2241::AID-IMMU2241>3.0.CO;2-E 10427987

[B41] Piccinni M. P. Scaletti C. Maggi E. Romagnani S. (2000). Role of hormone-controlled Th1- and Th2-type cytokines in successful pregnancy. J. Neuroimmunol. 109, 30–33. 10.1016/S0165-5728(00)00299-X 10969178

[B42] Pillai S. B. Rockwell L. C. Sherwood O. D. Koos R. D. (1999). Relaxin stimulates uterine edema via activation of estrogen receptors: blockade of its effects using ICI 182,780, a specific estrogen receptor antagonist. Endocrinology 140, 2426–2429. 10.1210/endo.140.5.6897 10218998

[B43] Plaks V. Birnberg T. Berkutzki T. Sela S. Benyashar A. Kalchenko V. (2008). Uterine DCs are crucial for decidua formation during embryo implantation in mice. J. Clin. Invest. 118, 3954–3965. 10.1172/jci36682 19033665 PMC2582932

[B44] Ponikowski P. Metra M. Teerlink J. R. Unemori E. Felker G. M. Voors A. A.. (2012). Design of the RELAXin in acute heart failure study. Am. Heart J. 163, 149–155. 10.1016/j.ahj.2011.10.009 22305830

[B45] Robertson S. A. (2007). Seminal fluid signaling in the female reproductive tract: lessons from rodents and pigs. J. Anim. Sci. 85, E36–E44. 10.2527/jas.2006-578 17085725

[B46] Robertson S. A. (2010). Immune regulation of conception and embryo implantation – all about quality control? J. Reprod. Immunol. 85, 51–57. 10.1016/j.jri.2010.01.008 20347158

[B47] Robertson S. A. Allanson M. Mau V. J. (1998). Molecular regulation of uterine leukocyte recruitment during early pregnancy in the mouse. Troph Res. 11, 101–120. 10.1016/s0143-4004(98)80009-x

[B48] Robertson S. A. Guerin L. R. Bromfield J. J. Branson K. M. Ahlstrom A. C. Care A. S. (2009). Seminal fluid drives expansion of the CD4+CD25+ T regulatory cell pool and induces tolerance to paternal alloantigens in mice. Biol. Reprod. 80, 1036–1045. 10.1095/biolreprod.108.074658 19164169 PMC2849830

[B49] Robertson S. A. Ingman W. V. O'Leary S. Sharkey D. J. Tremellen K. P. (2002). Transforming growth factor β – a mediator of immune deviation in seminal plasma. J. Reprod. Immunol. 57, 109–128. 10.1016/S0165-0378(02)00015-3 12385837

[B50] Robertson S. A. Mau V. J. Hudson S. N. Tremellen K. P. (1997). Cytokine-leukocyte networks and the establishment of pregnancy. Am. J. Reprod. Immunol. 37, 438–442. 10.1111/j.1600-0897.1997.tb00257.x 9228299

[B51] Robertson S. A. Mayrhofer G. Seamark R. F. (1992). Uterine epithelial cells synthesize granulocyte-macrophage colony-stimulating factor and interleukin-6 in pregnant and nonpregnant mice. Biol. Reprod. 46, 1069–1079. 10.1095/biolreprod46.6.1069 1391304

[B52] Robertson S. A. O'Leary S. Armstrong D. T. (2006). Influence of semen on inflammatory modulators of embryo implantation. Soc. Reprod. Fertil. Suppl. 62, 231–245. 16866321

[B53] Rodríguez-Martínez Kvist U. Ernerudh J. Sanz L. Calvete J. J. (2011). Seminal plasma proteins: what role do they play? Am. J. Reprod. Immunol. 66(Suppl. 1), 11–22. 10.1111/j.1600-0897.2011.01033.x 21726334

[B54] Rogers P. A. W. Murphy C. R. Squires K. R. MacLennan A. H. (1983). Effects of relaxin on the intrauterine distribution and antimesometrial positioning and orientation of rat blastocysts before implantation. J. Reprod. Fertil. 68, 431–435. 10.1530/jrf.0.0680431 6864659

[B55] Samuel C. S. Lin F. Hossain M. A. Zhao C. Ferraro T. Bathgate R. A.. (2007). Improved chemical synthesis and demonstration of the relaxin receptor binding affinity and biological activity of mouse relaxin. Biochemistry 46, 5374–5381. 10.1021/bi700238h 17425335

[B56] Samuel C. S. Tian H. Zhao L. Amento E. P. (2003). Relaxin is a key mediator of prostate growth and male reproductive tract development. Lab. Invest. 83, 1055–1067. 10.1097/01.LAB.0000079784.81186.B9 12861045

[B57] Schjenken J. E. Glynn D. J. Sharkey D. J. Robertson S. A. (2015). TLR4 signaling is a major mediator of the female tract response to seminal fluid in mice. Biol. Reprod. 93, 1–13. 10.1095/biolreprod.114.125740 26157066

[B58] Scott D. J. Layfield S. Riesewijk A. Morita H. Tregear G. W. Bathgate R. A. (2004). Identification and characterization of the mouse and rat relaxin receptors as the novel orthologues of human leucine-rich repeat-containing G-protein-coupled receptor 7. Clin. Exp. Pharmacol. Physiol. 31, 828–832. 10.1111/j.1440-1681.2004.04075.x 15566402

[B59] Sharkey D. J. Macpherson A. M. Tremellen K. P. Mottershead D. G. Gilchrist R. B. Robertson S. A. (2012a). TGF-β mediates proinflammatory seminal fluid signaling in human cervical epithelial cells. J. Immunol. 189, 1024–1035. 10.4049/jimmunol.1200005 22706080

[B60] Sharkey D. J. Tremellen K. P. Jasper M. J. Gemzell-Danielsson K. Robertson S. A. (2012b). Seminal fluid induces leukocyte recruitment and cytokine and chemokine mRNA expression in the human cervix after coitus. J. Immunol. 188, 2445–2454. 10.4049/jimmunol.1102736 22271649

[B61] Sherwood O. D. (1994). Relaxin, in The Physiology of Reproduction, eds Knobil E. Neill J. D. (New York, NY: Raven Press), 861–1009.

[B62] Siebel A. L. Gehring H. M. Reytomas I. G. Parry L. J. (2003). Inhibition of oxytocin receptor and estrogen receptor-alpha expression, but not relaxin receptors (LGR7), in the myometrium of late pregnant relaxin gene knockout mice. Endocrinology 144, 4272–4275. 10.1210/en.2003-0548 12959965

[B63] Snell G. D. (1941). Biology of the Laboratory Mouse. Philadelphia, PA: Blakiston.

[B64] Stults J. T. Bourell J. H. Canova-Davis E. Ling V. T. Laramee G. R. Winslow J. W.. (1990). Structural characterization by mass spectrometry of native and recombinant human relaxin. Biomed. Environ. Mass Spectrom. 19, 655–664. 10.1002/bms.1200191105 2076464

[B65] Taylor M. J. Clark C. L. (1994). Evidence for a novel source of relaxin: atrial cardiomyocytes. J. Endocrinol. 143, R5–R8. 10.1677/joe.0.143R005 7829984

[B66] Wira C. R. Grant-Tschudy K. S. Crane-Godreau M. A. (2005). Epithelial cells in the female reproductive tract: a central role as sentinels of immune protection. Am. J. Reprod. Immunol. 53, 65–76. 10.1111/j.1600-0897.2004.00248.x 15790340

[B67] Wood G. W. Hausmann E. H. Kanakaraj K. (1999). Expression and regulation of chemokine genes in the mouse uterus during pregnancy. Cytokine 11, 1038–1045. 10.1006/cyto.1999.0513 10623429

[B68] Zhao L. Roche P. J. Gunnersen J. M. Hammond V. E. Tregear G. W. Wintour E. M.. (1999). Mice without a functional relaxin gene are unable to deliver milk to their pups. Endocrinology 140, 445–453. 10.1210/endo.140.1.6404 9886856

