# Supplementary material for: Male Seminal Relaxin Contributes to Induction of the Post-mating Cytokine Response in the Female Mouse Uterus
Source: Front Physiol. 2017 Jun 19;8:422. doi: 10.3389/fphys.2017.00422 (PMC5474474; doi:10.3389/fphys.2017.00422)
Supplement: Supplementary file 1 [file Presentation1.PDF]

Supplemental Table 1 List of Antibodies

Peptide/Protein Target	Name of Antibody	Manufacturer / Cat. No.	Antibody Species Monoclonal/ Polyclonal	Dilution
Mouse CD45	TIB122	ATCC / cell culture supernatant	Rat monoclonal	1:1
Mouse Ly6G	RB6-8C5	BD-Biosciences Cat. 550291	Rat monoclonal	1:2
Rat IgG	Biotinylated anti-rat Ig secondary Ab	Dako E0468	Rabbit polyclonal	1:300

Supplemental Table 2

Oligonucleotide primers and conditions used for RT-PCR

gene	forward (5’-3’)	reverse (5’-3’)	size (bp)	annealing temp.
Agtr2	cagcctgcattttaaggagtgc	gctggtaatgtttctgctggtg	160	62
Ccl2	gcaccagccaactctcactg	ttgggatcatcttgctggtg	180	62
Cd80	agcctcgcttctcttggttg	cccgaaggtaaggctgttgt	365	62
Csf3	agtccctggagcaagtgagg	agagcctgcaggagaccttg	229	62
Cxcl10	catcctgctgggtctgagtg	aggctctctgctgtccatcc	322	62
Il6	tgggaaatcgtggaaatgag	gcattggaaattggggtagg	338	62
Il18	aaagaaagccgcctcaaac	gagagggtcacagccagtcc	348	62
Cxcl1	cttgaccctgaagctccctt	tgcacttcttttcgcacaac	369	60
Ptgs2	cccacttcaagggagtctgg	catctgctacgggaggaagg	195	62
Ptprc	ctcgtccactgcagagatgg	cagaaccattggcagcatgt	394	62
Smad3	gccccagagcaatattccag	ttccggttgacattggacag	327	62
Bcl2	catgtgtgtggagagcgtca	aggctgagcagggtcttcag	201	62
Rps27a	ccaggataaggaaggaattcctcctg	ccagcaccacattcatcagaagg	297	64
Rln	gtggatggacggattcattc'	aggcatttcagcgtcgtatc	192	66
Rxfp1	cagcctcactaaactgtacctc	tactgcaggaaataaaagtc	272	60
Tgfb1	cagacattcgggaagcagtg	cagccactcaggcgtatcag	176	60
Tgfb2	ctgtgcaggagtggcttcac	gcaggagatgtggggtcttc	231	60
Tgfb3	cctttcagcccaatggagac	tgaggatcaggtgtgggttg	150	60

Supplemental Figure 1

Real-time qRT-PCR quantification of *Rln, Tgfb1, Tgfb2, and Tgfb3* transcript levels in RNA extracted from testes (test.) and epididymides (epid.) from wild type and *Rln* (-/-; knockout; ko) sibling male mice, collected at 3 and 6 months (mo.) of age. For all *Tgfβ* isoforms, no significant effect of the relaxin gene deletion was evident. Histograms show relative transcript level normalized against *Rps27a* (mean ± SEM; n=3 individuals per tissue).

Supplemental Figure 2

Real-time qRT-PCR quantification of *Rln* (**A,E**), *Rxfp1*(**B,F**) or *Ptprc* (**C**) transcript levels in RNA extracted from the uteri of wild type mice treated or not with exogenous relaxin (Experiment 1; Panels **A** and **B**) or from the uteri of *Rln*+/+ and *Rln-/-* females (F) who had been mated with either *Rln-/- or Rln*+/+ males (M) (Experiment 2), as indicated (Panels **E** and **F**). Histograms show relative transcript level normalized against *Rps27a* (mean ± SEM; n=3 individuals per tissue). Panel D shows the quantification (mean ± SEM; n= 3 individuals per treatment, 12-20 frames per section) of percentage immunoreactive positivity in uterine sections from experiment 1; 'control'represents DAB staining in the absence of the primary antibodies; veh, vehicle treatment; relaxin, treatment with rhRLN.

Suppl. Fig.1

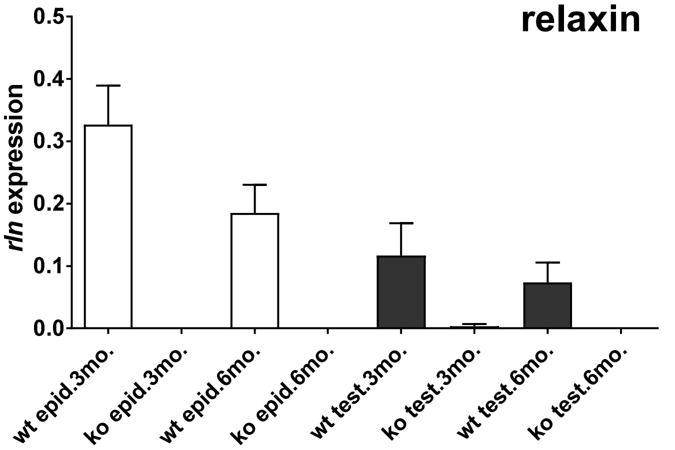

relaxin
rln expression
0.0
0.1
0.2
0.3
0.4
0.5
wt epid.3mo.
ko epid.3mo.
wt epid.6mo.
ko epid.6mo.
wt test.3mo.
ko test.3mo.
wt test.6mo.
ko test.6mo.

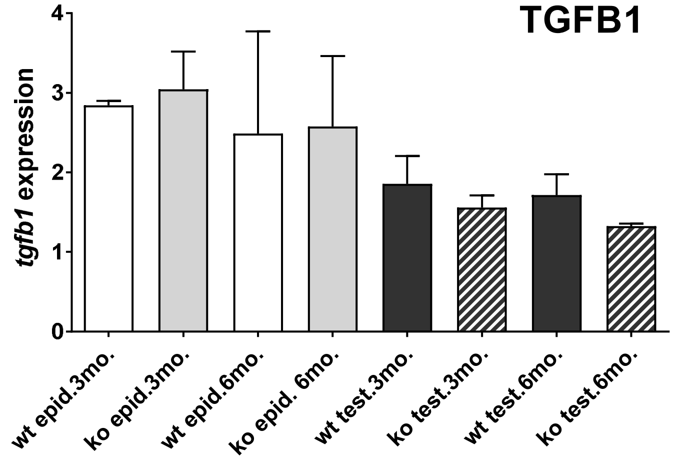

TGFB1
tgfb1 expression
0
1
2
3
4
wt epid.3mo.
ko epid.3mo.
wt epid.6mo.
ko epid. 6mo.
wt test.3mo.
ko test.3mo.
wt test.6mo.
ko test.6mo.

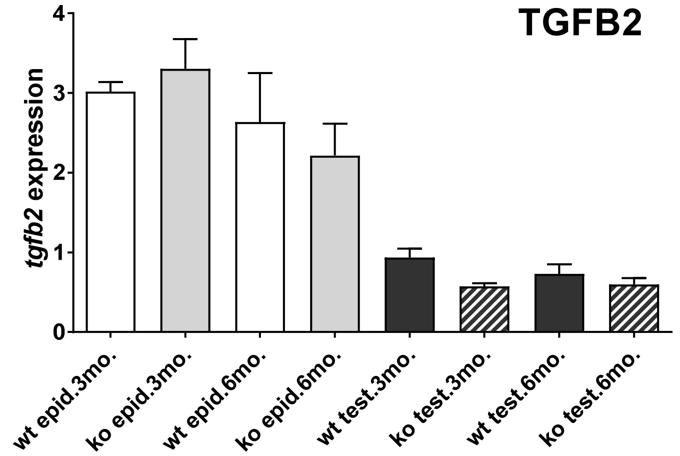

TGFB2
tgfb2 expression
0
1
2
3
4
wt epid.3mo.
ko epid.3mo.
wt epid.6mo.
ko epid.6mo.
wt test.3mo.
ko test.3mo.
wt test.6mo.
ko test.6mo.

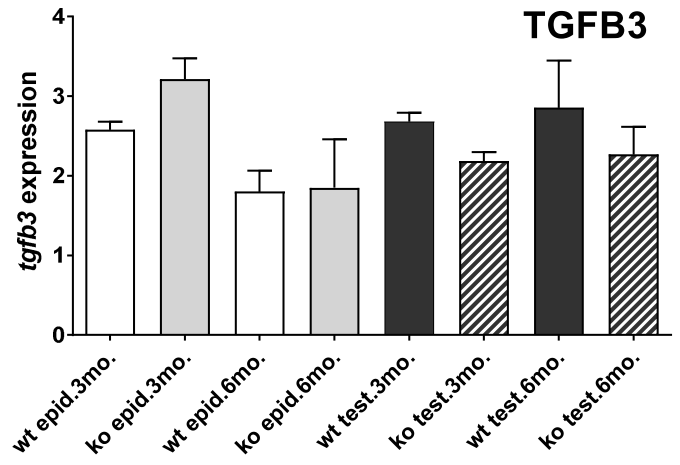

TGFB3
tgfb3 expression
0
1
2
3
4
wt epid.3mo.
ko epid.3mo.
wt epid.6mo.
ko epid.6mo.
wt test.3mo.
ko test.3mo.
wt test.6mo.
ko test.6mo.

Suppl. Fig. 2

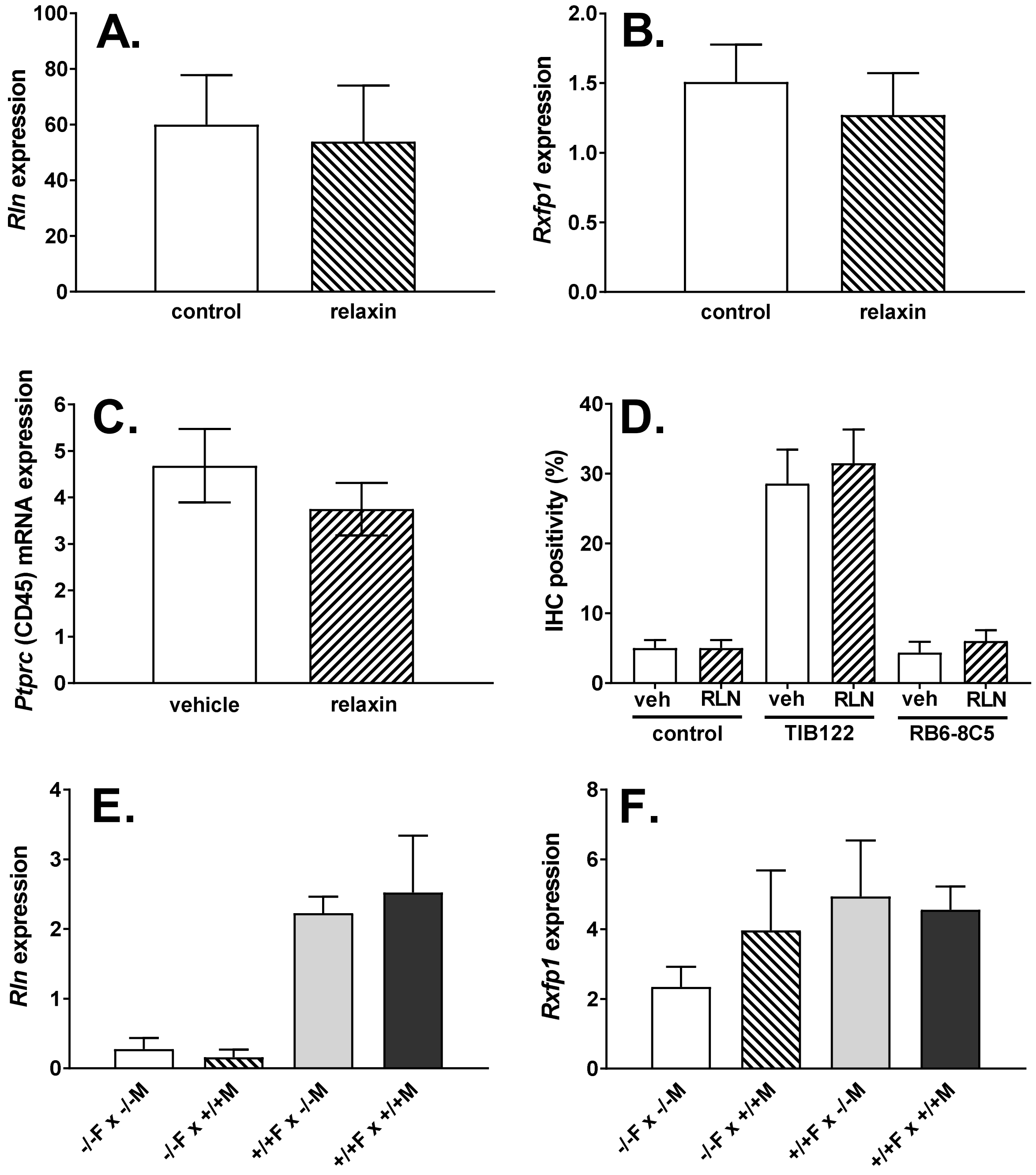
A.
Rln expression
100
80
60
40
20
0
control
relaxin
B.
Rxfp1 expression
2.0
1.5
1.0
0.5
0.0
control
relaxin
C.
Ptprc (CD45) mRNA expression
6
5
4
3
2
1
0
vehicle
relaxin
D.
IHC positivity (%)
40
30
20
10
0
veh
RLN
veh
RLN
veh
RLN
control
TIB122
RB6-8C5
E.
Rln expression
4
3
2
1
0
-/-F x -/-M
-/-F x +/+M
+/+F x -/-M
+/+F x +/+M
F.
Rxfp1 expression
8
6
4
2
0
-/-F x -/-M
-/-F x +/+M
+/+F x -/-M
+/+F x +/+M
